# Automatic Diagnosis of Mental Healthcare Information Actionability: Developing Binary Classifiers

**DOI:** 10.3390/ijerph182010743

**Published:** 2021-10-13

**Authors:** Meng Ji, Wenxiu Xie, Riliu Huang, Xiaobo Qian

**Affiliations:** 1 School of Languages and Cultures, University of Sydney, Sydney 2006, Australia; rhua5035@uni.sydney.edu.au; 2 Department of Computer Science, City University of Hong Kong, Hong Kong 999077, China; Vasiliky@outlook.com; 3 School of Computer Science, South China Normal University, Guangzhou 510631, China; xiaoboqian1221@outlook.com

**Keywords:** mental healthcare, information quality assessment, actionability, binary classification, natural language features

## Abstract

We aimed to develop a quantitative instrument to assist with the automatic evaluation of the actionability of mental healthcare information. We collected and classified two large sets of mental health information from certified mental health websites: generic and patient-specific mental healthcare information. We compared the performance of the optimised classifier with popular readability tools and non-optimised classifiers in predicting mental health information of high actionability for people with mental disorders. sensitivity of the classifier using both semantic and structural features as variables achieved statistically higher than that of the binary classifier using either semantic (*p* < 0.001) or structural features (*p* = 0.0010). The specificity of the optimized classifier was statistically higher than that of the classifier using structural variables (*p* = 0.002) and the classifier using semantic variables (*p* = 0.001). Differences in specificity between the full-variable classifier and the optimised classifier were statistically insignificant (*p* = 0.687). These findings suggest the optimised classifier using as few as 19 semantic-structural variables was the best-performing classifier. By combining insights of linguistics and statistical analyses, we effectively increased the interpretability and the diagnostic utility of the binary classifiers to guide the development, evaluation of the actionability and usability of mental healthcare information.

## 1. Introduction

Information readability and actionability form two key factors of the effectiveness of patient-oriented healthcare information [1,2,3,4,5]. Many current online healthcare information quality evaluations focus on readability assessment. This benefits from the long tradition of quantitative readability evaluation in medical and health education using readability instruments [6,7,8,9,10]. Even though the two concepts are distinct from each other in both research and clinical practice, many current studies still confuse and conflate the two distinct concepts into a single dimension of health information quality assessment. Actionable content means information that can automatically prompt the best decisions about care at the point in time when clinical decisions need to be made [11,12,13]. This requires the design of health information that is based on the real-life circumstances of the intended information users and best reflects their practical needs, varying health literacy levels, cognitive abilities, socioeconomic circumstances, and other determinants [14,15,16,17]. Actionability assessment thus requires a distinct approach to readability or understandability assessment. Actionability can have an important impact on the acceptability and practical usability of the information for target readers [18,19]. Existing approaches to the evaluation of health information actionability are often qualitative. Patient Education Materials Assessment Tool (PEMAT) is widely used by health and medical professionals to evaluate the practical usability of printed or audio-visual materials [20,21]. However, there is a lack of quantitative tools to assist health professionals with the evaluation of the actionability of mental health resources.

Our study aimed to offer an automatic quantitative assessment of mental health information actionability. This was based on two major types of online mental healthcare information: generic and patient-specific. Generic mental health information is written for the general public, with an accessible, understandable language, but without specifying the intended patients or information users. This represents the mainstream health information in many medical and health domains. Another major type of mental healthcare information is patient-specific, using a similarly easy, plain, and understandable language but with a focus on well-defined patients and reader groups. General mental health information focuses on the explanation of symptoms, signs of prevalent mental disorder, wide-ranging causes, and determinants, as well as generic treatment plans and interventions. By contrast, patient-specific mental health information often adopts a narrative communication style, discussing the diverse, complex yet recurrent practical needs of the intended readers, recognising their potential for achieving better mental health and wellbeing, adapting general treatment plans and interventions to suit their needs, and taking into consideration likely barriers to external resources. An increasing number of not-for-profit organisations in English-speaking countries are actively engaged in the development of patient-specific mental health information for vulnerable people [22,23,24]. This increasing direction towards personalised mental healthcare support through patient-tailored mental health information provides valuable resources to the development of automatic assessment instruments, which, in turn, will further advance research and improve clinical practice in patient-centred mental healthcare.

## 2. Methods

### 2.1. Information Sources, and Search Strategies

We started the search for generic and patient-specific mental health resources on Google. We limited the search to the 100 top websites on mental healthcare up to 1 July 2021. We used the label of HON.Net as a measure of health and medical content quality control [25]. With the collection of generic mental healthcare information, 36 websites were excluded for not having been certified by HON.Net, 22 websites were excluded for not addressing general readers, and 6 were excluded for not being suitable for the automatic statistical analysis based on natural language features, such as too short paragraphs, sentences, or passages. With patient-specific mental healthcare information, 36 websites were excluded for not having been certified by HON.Net, 42 websites were excluded for not addressing general readers, and 4 were excluded for not being suitable for natural language mining and subsequent quantitative assessments. In the final screened texts, we randomly selected 70% from each text group to develop the training and testing dataset. The final dataset of patient-specific information included mental health selfcare resources for 4 population groups: teenagers (aged 11–18) (8.9%), young adults (aged 18–35) (87.5%), people over 65 years (1.67%), women (1.17%), and men (0.76%).

The two sets of mental healthcare information collected, that is, generic (GEN) and patient-specific (PAS), were fully annotated with two sets of natural language features using Readability Studio (Oleander Software) [26,27] and the English semantic annotation system (USAS) developed by the University of Lancaster, United Kingdom [28,29,30]. Readability Studio annotated the health information with rich structural features to help us measure the lexical, morphological, and syntactic complexity of the texts. Some of these features were studied extensively in the field of readability assessment, such as the average number of characters, average number of syllables, number of monosyllabic words, average number of sentences per paragraph, average sentence length, whereas others were less studied. Structural features added by Readability Studio were collectively labelled with TOF in the training and testing of machine learning classifiers. The automatic semantic annotation with USAS added rich semantic information of the mental healthcare resources.

### 2.2. Semantic Feature Labelling Strategy

Semantic features were well studied in medical document classification and clinically significant information retrieval. In our study, semantic features (labelled as SOF) refer to health information contents. The semantic classification of USAS was loosely based on Tom McArthur’s Longman Lexicon of Contemporary English, which broadly divided the English lexicon into 22 large categories (Table 2) [31,32,33]. Semantic annotation has wide applications in medical and health informatics, such as document classification and information retrieval. In the clinic, the semantic annotation has been explored to organise unstructured clinical information or data to support medical research or clinical trials. It can aid in the automatic extraction of critical information from clinical texts such as temporal information, symptoms, diseases to facilitate clinical decision making. Compared to structural features, semantic features can help with more contextualised analyses of health information, for example, in our study, this means how the use of certain semantic classes (Table 2) such as medical and health terms (B), emotions (E), sports (K), movement M), measurements (N), temporal expressions (T), psychological actions, states and processes (X), science and technology (Y), and proper names (Z), may affect the levels of the actionability of mental healthcare resources.

Since the USAS semantic annotation scheme was designed for general language studies, we adapted the descriptive labels of some semantic categories (notably B, K, Z) to reflect its application in the study of mental healthcare information. Subcategories of the original 22 large semantic categories that occurred rarely in mental healthcare information were trimmed. As a result, the adjusted descriptive labels indicated the largest and most frequent subcategory within each large semantic category. For example, the original USAS descriptive label for B was (Body and the Individual). In our study, it was found that most of the subclasses of B belonged to B1 (Anatomy and physiology); B2 (Health and disease); B3 (Medicines and medical treatment). There were large missing cases of B4 (Cleaning and personal care) and B5 (Clothes and personal belongings). We, therefore, adapted B to Medicine and Health terms in our study.

The original USAS descriptive label for K was Entertainment, which includes K1 (Entertainment generally), K2 (Music and related activities), K3 (Recorded sound), K4 (Drama, the theatre and show business), K5 (Sports and games generally) and K6 (Children’s games). Similarly, the original descriptive label of category I was (Money), including I1 (Money generally), I2 (Business), I3 (work and employment), and I4 (Industry). The most frequent subcategory of mental healthcare resources was I3 on employment, so we relabelled I as Work Employment. Most words of K belonged to K5 and K6; we, thus, adjusted the descriptive label to reflect the most relevant subcategories of K in our study on mental healthcare resources, especially for youth mental healthcare. Another example is the semantic category Z, whose original USA descriptive label was Names (Z1–Z3) and Grammar (Z4–Z9 plus Z99). To distinguish the effects of infrequent expressions, such as medical jargon, we separated Z99 (out-of-dictionary expressions) from other Z subclasses: Z1 (personal names); Z2 (geographical names); Z3 (other proper names), Z5 (grammatical bins), Z6 (negative expressions), Z7 (if), Z8 (pronouns), and Z9 (trash can).

### 2.3. Statistics

#### 2.3.1. Readability Assessment

Table 1 shows the statistical analysis (Mann–Whitney U test of 2 independent samples) of the distribution of structural features (TOF) in general, non-specified and patient-specific mental healthcare information. First, we calculated the overall difficulty of written mental health information using validated readability assessment tools including Flesch Reading Ease Score, FORCAST, Gunning Fog Index, SMOG Index. The mean Flesch Ease Reading score of generic, non-specified mental healthcare information was 46.71 (SD = 11.92) and was statistically more difficult than that of patient-specific mental health information (mean = 66.74, SD = 12.27, Mann–Whitney U test, *p* = 0.000). This suggests that generic information with a mean of 46.61 was suitable for college students (Flesch Reading Ease range: 50–30); and patient-tailored mental health information could be easily understood by 13–15-year-old students or general readers with Year 9 education (Flesch Reading Ease range: 70–60). The average Gunning Fog score of the generic mental health information was 12.46 (SD = 2.03) and was statistically higher than that of patient-tailored mental health information (mean = 9.02, SD = 1.93, *p* = 0.000). The difficulty of generic mental health information measured by SMOG Index score was 12.50 (SD = 1.50) and was statistically higher than patient-tailored resources (mean = 10.12, SD = 1.6, *p* = 0.000). Both results of the Gunning Fog Index and the SMOG Index suggested that patient-tailored information was suitable for readers with Year 9–10 education, and generic mental healthcare resources required at least three more years of education. Both generic and patient-specific mental healthcare information was above the Year 6–8 reading levels recommended by the World Health Organisation.

#### 2.3.2. Statistical Differences between Patient and Generic Health Information

Lexical complexity was measured by 10 structural features including medical jargons, number of unique words, repeated words, article mismatches, redundant phrases, overused words, wordy items, cliché, number of proper nouns and number of numerals. The mean of 3 lexical features of generic mental healthcare information was statistically similar to that of patient-specific information: repeated words (mean__GEN_ = 0.02, mean_ _PAS_ = 0.02, *p* = 0.732), redundant phrases (mean__GEN_ = 0.16, mean_ _PAS_ = 0.15, *p* = 0.945), and cliches (mean__GEN_ = 0.12, mean_ _PAS_ = 0.12, *p* = 0.685). For the remaining 7 structural features measuring lexical complexity, the mean of non-specified mental healthcare information was statistically higher than that of patient-specific mental healthcare information: medical jargons (mean__GEN_ = 11.25, mean_ _PAS_ = 5.14, *p* = 0.000), number of unique words (mean__GEN_ = 426.45, mean_ _PAS_ = 329.19, *p* = 0.000), article mismatches (mean__GEN_ = 0.03, mean_ _PAS_ = 0.03, *p* = 0.000), overused words (mean__GEN_ = 16.49, mean_ _PAS_ = 9.97, *p* = 0.000), wordy items (mean__GEN_ = 42.24, mean_ _PAS_ = 18.41, *p* = 0.000), number of proper nouns (mean__GEN_ = 26.05, mean_ _PAS_ = 16.74, *p* = 0.000), and number of numerals (mean__GEN_ = 6.7, mean_ _PAS_ = 6.05, *p* = 0.000).

In terms of morphological complexity, the mean of generic mental healthcare information was statistically higher than that of patient-specific information in 7 categories: average number of characters (mean__GEN_ = 5.16, mean_ _PAS_ = 4.64, *p* = 0.000), average number of syllables (mean__GEN_ = 1.73, mean_ _PAS_ = 1.50, *p* = 0.000), number of monosyllabic words (mean__GEN_ = 660.40, mean_ _PAS_ = 620.35, *p* = 0.000), number of complex (3+ syllable) words (mean__GEN_ = 218.95, mean_ _PAS_ = 114.68, *p* = 0.000), number of unique 3+ syllable words (mean__GEN_ = 127.2, mean_ _PAS_ = 67.81, *p* = 0.000), number of long (6+ characters) words (mean__GEN_ = 434.95, mean_ _PAS_ = 283.58, *p* = 0.000), and number of unique long words (mean__GEN_ = 245.87, mean_ _PAS_ = 157.58, *p* = 0.000). The mean of the number of unique monosyllabic words between two sets of mental healthcare information was statistically similar (mean__GEN_ = 162.4, mean_ _PAS_ = 157.86, *p* = 0.091).

Lastly, generic patient mental health information was syntactically more complex than patient-specific mental healthcare information: number of difficult sentences (more than 22 words) (mean__GEN_ = 14.69, mean_ _PAS_ = 8.68, *p* = 0.000), average sentence length (mean__GEN_ = 13.8, mean_ _PAS_ = 12.73, *p* = 0.000), and passive voice (mean__GEN_ = 5.22, mean_ _PAS_ = 2.79, *p* = 0.000). Characteristics of syntactical structures of patient-specific mental healthcare information included the use of more lengthy, descriptive paragraphs compared to syntactic brevity of generic, non-specified mental health information: average number of sentences per paragraph (mean__GEN_ = 1.54, mean_ _PAS_ = 2.58, *p* = 0.000); stronger emphasis on logical coherence: sentences that begin with conjunctions (and, but, though, while, even though, etc.) (mean__GEN_ = 1.11, mean_ _PAS_ = 1.64, *p* = 0.000); and the use of more interactive sentence structures: number of interrogative sentences (questions) (mean__GEN_ = 2.81, mean_ _PAS_ = 4.3, *p* = 0.000) and number of exclamatory sentences (mean__GEN_ = 0.08, mean_ _PAS_ = 1.35, *p* = 0.000).

Table 2 shows that the mean of generic, non-specified mental healthcare information was statistically higher than that of patient-specific mental healthcare information in semantic categories of general and abstract terms (A) (mean__GEN_ = 236.46, mean_ _PAS_ = 192.704, *p* = 0.000), medical and health expressions (B) (mean__GEN_ = 83.122, mean_ _PAS_ = 36.17, *p* = 0.000), emotions (E) (mean__GEN_ = 40.559, mean_ _PAS_ = 27.789, *p* = 0.000), food and beverage (F) (mean__GEN_ = 8.794, mean_ _PAS_ = 6.583, *p* = 0.004), government and politics (G) (mean__GEN_ = 3.421, mean_ _PAS_ = 3.313, *p* = 0.000), dwelling and housing (H) (mean__GEN_ = 3.479, mean_ _PAS_ = 3.071, *p* = 0.004), work and employment (I) (mean__GEN_ = 9.363, mean_ _PAS_ = 8.121, *p* = 0.000), sports and games (K) (mean__GEN_ = 4.9, mean_ _PAS_ = 4.176, *p* = 0.002), living things (L) (mean__GEN_ = 5.711, mean_ _PAS_ = 4.389, *p* = 0.000), measurements (N) (mean__GEN_ = 62.396, mean_ _PAS_ = 44.089, *p* = 0.000), general substances (O) (mean__GEN_ = 16.960, mean_ _PAS_ = 13.138, *p* = 0.000), education (P) (mean__GEN_ = 4.614, mean_ _PAS_ = 4.290, *p* = 0.000), social actions, states and processes (S) (mean__GEN_ = 73.062, mean_ _PAS_ = 72.781, *p* = 0.026), temporal expressions (T) (mean__GEN_ = 37.985, mean_ _PAS_ = 34.453, *p* = 0.000), environment (W) (mean__GEN_ = 1.879, mean_ _PAS_ = 1.268, *p* = 0.000), psychological actions, states and processes (X) (mean__GEN_ = 67.294, mean_ _PAS_ = 62.999, *p* = 0.020), science and technology (Y) (mean__GEN_ = 2.69, mean_ _PAS_ = 2.59, *p* = 0.001), proper names and grammar (Z) (mean__GEN_ = 389/823, mean_ _PAS_ = 359.010, *p* = 0.000) and out-of-dictionary expressions (Z99) (mean__GEN_ = 33.909, mean_ _PAS_ = 16.810, *p* = 0.000).

#### 2.3.3. Feature Optimization Using Principal Component Analysis

**Figure 1:**
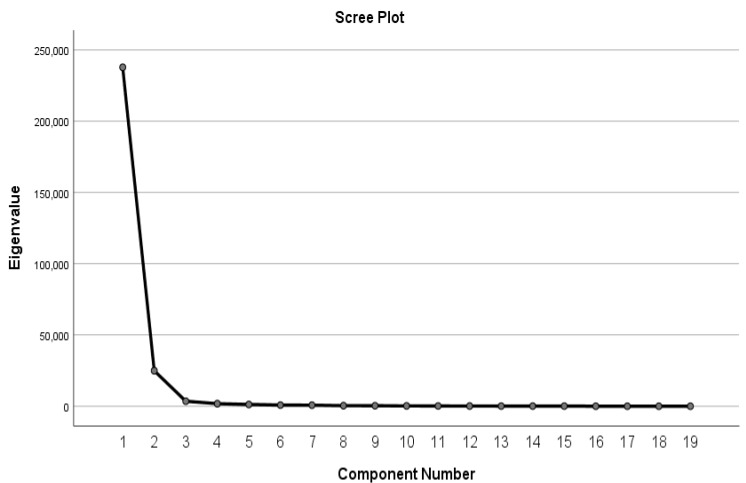
Factor Analysis Scree Plot.

We divided the entire dataset between 70% for constructing the binary classifier and 30% for validating the classifier. Table 3 shows the result of exploratory factor analysis (EFA) used to reduce the dimensions of observed variables, i.e., the total 49 natural language features. Within the two-dimensional instrument constructed, the first and second dimensions accounted for 42.361% and 36.396% of the total variance in the 70% training dataset, respectively. Figure 1 is the screen plot that visualised that increases in the amount of variance explained by the instrument started to flatten after the second dimension, again suggesting that the optimised dimension number be set at 2.

Table 4 exhibits the rotated loading (varimax rotation with Kaiser normalisation) of the observed variables on the first two large dimensions. The first dimension or component of the instrument encompassed 9 structural features and 1 semantic feature (B medicine/health terms). Seven variables had medium effect sizes (Hedges’g 0.5–0.8), which included: number of unique words (Hedges’g = 0.601, CLES = 0.665), overused words (Hedges’g = 0.548, CLES = 0.651), number of long (6+ characters) words (Hedges’g = 0.647, CLES = 0.676), number of difficult sentences (more than 22 words) (Hedges’g = 0.643, CLES = 0.675), passive voice (Hedges’g = 0.549, CLES = 0.651), medicine/health B (Hedges’g = 0.76, CLES = 0.704), and out of dictionary Z99 (Hedges’g = 0.669, CLES = 0.682). Four variables of large effect size (Hedges’g > 0.8) included: wordy items (Hedges’ g = 1.002, CLES = 0.761), number of complex (3+ syllable) words (Hedges’g = 0.879, CLES = 0.733), number of unique 3+ syllable words (Hedges’g = 0.97, CLES = 0.775), and number of unique long words (Hedges’g = 0.763, CLES = 0.729). The first dimension of variables summarised 10 variables measuring the morphological, lexical, syntactic complexity of mental healthcare resources, whereas the second dimension highlighted 9 out of the original 22 semantic variables, which were correlated with each other in the distribution in the 70% training data.

## 3. Results

After exploratory factor analysis on the 70% training data, we validated the logistic regression model on the remaining 30% dataset and compared the performance of the optimised binary classifier with that of popular readability tools and binary classifiers with original variables. The 4 binary classifiers were based on semantic variables (22), structural variables (27), both semantic and structural features (49), and the optimised variables (19) through exploratory factor analysis. Table 5 shows the paired-sample Wilcoxon signed-rank test, which assessed whether the difference between the AUC of each classifier or readability tool and the reference 0.5 was statistically significant or not. A p smaller than 0.05 was considered statistically significant. It shows the AUC of readability tools and binary classifiers using different variables was statistically higher than the reference AUC.

Table 6 shows the paired-sample *t*-test of the AUC of readability formula and binary classifiers using different variables, with the adjusted Bonferroni significance at 0.00179. *p*-values smaller than 0.00179 were considered statistically significant. It shows that among the 4 readability formula, Gunning Fog Index (AUC = 0.893) and Flesch Reading Index (*p* = 0.882) were the two top classifiers and the differences in their AUC was statistically insignificant (*p* = 0.009852 > 0.00179). Among the four binary classifiers, the two top classifiers were the one using all variables (49), including semantic and structural (AUC = 0.872) and the one based on optimised variables (19 variables) (AUC = 0.863), and the difference between the two was statistically insignificant (*p* = 0.2292). The AUC of Gunning Fog Index was statistically higher than the binary classifier using 49 variables (*p* = 0.000168) and the optimised binary classifier using 19 variables (*p* = 0.000462). The AUC of the Flesch Reading Ease Index was statistically similar to that of the binary classifier using 49 variables (*p* = 0.02513) and the optimised binary classifier using 19 variables (*p* = 0.1837).

Table 7 shows that despite the sensitivity and specificity pairs of the 2 top readability formula-based classifiers: Gunning Fog Index and the Flesch Reading Ease Index. It shows that under the different thresholds, sensitivity increases as specificity decreases. Table 8 shows the sensitivity and specificity of 4 binary classifiers using natural language features as variables. When setting the specificity at 0.85, the binary classifier with all variables (49) had the highest sensitivity (0.907), followed by the optimised classifier (19) using exploratory factor analysis (0.890), Gunning Fog (0.769), and Flesch Reading Ease (0.729). As a result, the two binary classifiers had achieved better sensitivity and specificity when compared to the readability formula-based classifiers, which had much lower specificity.

Table 9 shows the paired-sample *t*-test of differences in sensitivity between 4 binary classifiers using natural language features as independent variables. *p*-values are statistically significant when smaller than the Bonferroni correction (adjusted alpha = 0.00833). It shows that sensitivity of the classifier using both semantic and structural features (49) as variables achieved statistically higher than that of the binary classifier using either semantic (22) (*p* = 0.000) or structural features (27) (*p* = 0.0010). Similarly, the sensitivity of the binary classifier using optimised features (factor analysis) (19) was statistically higher than that of the binary classifier using either semantic (*p* = 0.000) or structural features (*p* = 0.0020). The difference in sensitivity between the binary classifier using full variable set (49) and the optimised classifier (19) was statistically insignificant (*p* = 0.0398 > 0.00833).

Table 10 shows the paired-sample *t*-test of differences in specificity between 4 binary classifiers. It shows that the specificity of the classifier using full variables (49) was statistically higher than that of the classifier based on structural variables (27) (*p* = 0.001) but statistically similar to that of the classifier based on semantic variables (22) (*p* = 0.011 > 0.00833). The specificity of the optimised classifier was statistically higher than that of the classifier using structural variables (27) (*p* = 0.002) and the classifier using semantic variables (22) (*p* = 0.001). Differences in specificity between the full-variable classifier and the optimised classifier were statistically insignificant (*p* = 0.687). These findings suggest that the optimised classifier using as few as 19 semantic-structural variables was the best-performing classifier.

## 4. Discussions

Improving the quality and usability of current mental healthcare necessitates the development of highly actionable and better-targeted resources for people with different mental disorders or at high risks of developing mental diseases. This requires a more personalised and patient-centred approach to mental health information evaluation. The increasing amount of high-quality mental health information on the internet provides valuable first-hand materials to develop new quantitative evaluation tools and systems. Our study has made a useful attempt in this direction. In the development of automatic tools for the evaluation of mental health information actionability, we found that semantic features had an important role in actionability on mental health resources. For example, there were three semantic categories in which the mean of patient-specific mental healthcare information was statistically higher than that of generic mental health information: arts and cultures (C) (mean__GEN_ = 33.909, mean_ _PAS_ = 16.810, *p* = 0.000); locations (M) (mean__GEN_ = 33.909, mean_ _PAS_ = 16.810, *p* = 0.000), and speech acts (Q) (mean__GEN_ = 33.909, mean_ _PAS_ = 16.810, *p* = 0.000). These contrast findings suggested that generic information was richer and more varied compared to patient-specific mental healthcare information. Discussions in generic mental healthcare covered a broad range of risk factors causing mental disorders the public, such as social and political circumstances (G, S), environmental stressors (W), household and living environments (H), employment (I), nutrition (F), individual attributes (E), physical activities (K), science, technology, and medicine (B, Y), and education (P). The wide range of topics in general mental healthcare information, despite being more informative than patient-specific mental healthcare resources, could significantly reduce the actionability of the health information. By contrast, patient-specific mental healthcare information had stronger focuses on more concrete and tangible approaches to mental healthcare such as artistic and creative activities, as typical words in the semantic category C were artwork, caricature, carvings, crochet, D.I.Y. graphics, knit, paintbrush, photos, and paintings. Patient-specific mental health information also had a statistically higher (*p* = 0.045) use of speech acts expressions such as address, (have) conversation (about your mental health), tell (their stories), (your) point of contact, question, speak (with your communities), (peer) mentoring (programmes), talk (about your mental health openly), ask (what happened?), and talk (with a friend in need).

In our study, since most structural-semantic features had statistically significant differences (*p* = 0.000) in the two sets of mental healthcare information, we computed the adjusted effect size Hedges’g (for 2 independent samples of non-equal sizes) and common language effect sizes (CLES) measures. The general rules of thumb described by Cohen suggest that an effect size of 0.2 represents a “small” effect, an effect size of 0.5 represents a “medium” effect, and an effect size of 0.8 represents a “large” effect. CLES ranges between 0 and 1 and has a positive correlation with effect sizes. We found that an important shared property of structural features, which were retained in the variable reduction process, were those with medium or large effect sizes, suggesting that effect sizes can be a useful indicator of whether a certain observed variable is suitable to discriminate binary-dependent variables. Specifically, structural-semantic variables that had medium effect (Hedges’g 0.5–0.8) and eventually included in the optimised classifier included medical jargon (Hedges’ g = 0.585, CLES = 0.661), number of unique words (Hedges’g = 0.601, CLES = 0.665), overused words (Hedges’g = 0.548, CLES = 0.651), number of long (6+ characters) words (Hedges’g = 0.647, CLES = 0.676), number of difficult sentences (more than 22 words) (Hedges’g = 0.643, CLES = 0.675), passive voice (Hedges’g = 0.549, CLES = 0.651), Medicine/Health (B) (Hedges’g = 0.76, CLES = 0.704), Out of Dictionary (Z99) (Hedges’g = 0.669, CLES = 0.682). Variables of a large effect size (Hedges’g > 0.8) and included in the optimised classifier were wordy items (Hedges’ g = 1.002, CLES = 0.761), number of complex (3+ syllable) words (Hedges’g = 0.879, CLES = 0.733), number of unique 3+ syllable words (Hedges’g = 0.97, CLES = 0.775), and number of unique long words (Hedges’g = 0.763, CLES = 0.729). The linguistic meanings and the varying discriminating functionality of these structural and semantic features as measured by their statistical significance (*p* < 0.05) and effect sizes (Hedges’g > 0.5) were used to develop automatic binary classifiers to assess the actionability of mental healthcare information.

Study Limitation: Mental health is highly complex. Patients with different demographic profiles and mental disorders need well-designed resources to better support them. In our study, we divided mental health information into patient-oriented and generic mental health information. However, within patient-oriented health information, our newly developed quantitative tool could not assess whether a certain piece of mental health information is more suitable for a particular social group, such as young people, elderly people or adults, men, or women. There is space to develop evaluation tools to support health information assessment for specific populations.

Future Work: Increasing the actionability of mental health information for needed populations can significantly improve the quality of current mental health services in both developed and developing countries. To achieve this goal, the development of quantitative and machine learning-based evaluation tools and instruments will provide healthcare providers with much-needed resources. In our study, we made a useful attempt towards this goal. In future work, we aim to enrich the contents of the classifiers by testing different sets of language features such as sentiment features [34,35]. We also aim to test our methods with mental health resources in languages other than English to help better support mental health organizations working with multicultural, multilingual populations. These useful tools, such as the one we developed, do not assume any prior knowledge of the patients’ languages, which could effectively close the language gap between patients and health professionals supporting them.

## 5. Conclusions

Our study developed a quantitative instrument to assist with the automatic evaluation of the actionability of mental healthcare information. By combining the insights of linguistics and statistical analyses, we effectively increased the interpretability and the diagnostic utility of the binary classifiers to guide the development and evaluation of the actionability and usability of mental healthcare information.

## Figures and Tables

**Table 1:** Mann–Whitney U test, Effect Sizes (*d_CoheN_*) and Common language effect sizes (CLES) of structural features (TOF).

Structural Language Features (TOF)	PAS		GEN		Mann–Whitney U		d_Cohen_		CLES
	Mean	Std. Deviation	Mean	Std. Deviation	Z	P	*g_Hedges_*	95% C.I	
Readability Measurements									
Flesch Reading Ease	66.74	12.27	46.71	11.92	−27.94	0.000	−1.659	−1.766, −1.551	0.880
FORCAST	9.94	0.93	11.30	0.82	−26.705	0.000	1.562	1.456, 1.668	0.865
Gunning Fog Index	9.02	1.93	12.46	2.03	−28.708	0.000	1.732	1.623, 1.841	0.890
SMOG Index	10.12	1.60	12.50	1.50	−26.710	0.000	1.540	1.435, 1.646	0.862
Lexical Complexity									
Medical jargons	5.14	7.84	11.25	12.12	−15.490	0.000	0.585	0.49, 0.68	0.661
Number of unique words	329.19	148.56	426.45	171.48	−13.374	0.000	0.601	0.506, 0.697	0.665
Repeated words	0.02	0.16	0.02	0.14	−0.342	0.732	0.000	−0.093, 0.093	0.500
Article mismatches	0.03	0.39	0.03	0.19	−2.278	0.023	0.000	−0.093, 0.093	0.500
Redundant phrases	0.15	0.44	0.16	0.59	−0.068	0.945	0.019	−0.074, 0.112	0.505
Overused words	9.97	11.03	16.49	12.56	−15.794	0.000	0.548	0.453, 0.642	0.651
Wordy items	18.41	17.82	42.24	27.64	−22.941	0.000	1.002	0.903, 1.101	0.761
Cliché	0.12	0.42	0.12	0.40	−0.405	0.685	0.000	−0.093, 0.093	0.500
Number of proper nouns	16.74	27.37	26.05	27.88	−11.990	0.000	0.337	0.243, 0.43	0.594
Number of numerals	6.05	12.22	6.70	12.07	−8.593	0.000	0.054	−0.039, 0.147	0.515
Morphological Complexity									
Average number of characters	4.64	0.35	5.16	0.32	−27.139	0.000	1.558	1.452, 1.664	0.865
Average number of syllables	1.50	0.14	1.73	0.14	−27.329	0.000	1.643	1.536, 1.75	0.877
Number of monosyllabic words	620.35	419.24	660.40	382.71	−3.852	0.000	0.100	0.007, 0.193	0.528
Number of unique monosyllabic words	157.86	56.43	162.40	58.75	−1.688	0.091	0.079	−0.014, 0.172	0.522
Number of complex (3+ syllable) words	114.68	96.87	218.95	133.45	−20.794	0.000	0.879	0.782, 0.977	0.733
Number of unique 3+ syllable words	67.81	46.31	127.20	61.92	−22.513	0.000	1.070	0.97, 1.169	0.775
Number of long (6+ characters) words	283.58	210.03	434.95	251.32	−16.123	0.000	0.647	0.552, 0.743	0.676
Number of unique long words	157.58	90.18	245.87	111.59	−18.629	0.000	0.860	0.763, 0.957	0.729
Syntactic Complexity									
Average number of sentences per paragraph	2.58	8.09	1.54	0.36	−7.647	0.000	−0.193	−0.286, −0.099	0.554
Number of difficult sentences (more than 22 words)	8.68	7.88	14.69	10.37	−14.847	0.000	0.643	0.548, 0.738	0.675
Average sentence length	12.73	2.82	13.80	2.93	−8.281	0.000	0.371	0.278, 0.465	0.604
Passive voice	2.79	3.85	5.22	4.84	−15.280	0.000	0.549	0.454, 0.644	0.651
Sentences that begin with conjunctions	1.64	2.67	1.11	1.88	−4.817	0.000	−0.234	−0.327, −0.141	0.566
Number of interrogative sentences (questions)	4.30	4.70	2.81	4.46	−10.005	0.000	−0.326	−0.42, −0.233	0.591
Number of exclamatory sentences	1.35	3.35	0.08	0.40	−15.709	0.000	−0.564	−0.658, −0.469	0.655

**Table 2:** Mann–Whitney U test, Effect Sizes (*d_CoheN_*) and CLES of semantic features (SOF).

Semantic Language Features (SOF)	PAS		GEN		Mann–Whitney U		d_Cohen_		CLES
	Mean	Std. Deviation	Mean	Std. Deviation	Z	P	*g_Hedges_*	95% C.I	
A General/abstract terms	192.704	136.215	236.460	137.767	−8.986	0.000	0.319	0.226, 0.413	0.589
B Medicine/Health	36.170	48.766	83.122	70.517	−20.341	0.000	0.76	0.663, 0.856	0.704
C Arts and Culture	1.086	2.177	0.834	1.892	−2.320	0.020	−0.125	−0.218, −0.031	0.535
E Emotion	27.789	24.826	40.559	31.436	−10.517	0.000	0.445	0.351, 0.539	0.624
F Food	6.583	17.441	8.794	22.006	−2.900	0.004	0.11	0.017, 0.203	0.531
G Government	3.313	6.211	3.421	6.271	−2.986	0.003	0.017	−0.076, 0.11	0.505
H Dwelling	3.071	4.797	3.479	5.305	−4.073	0.000	0.08	−0.013, 0.173	0.523
I Employment	8.121	17.709	9.363	13.094	−8.108	0.000	0.081	−0.012, 0.174	0.523
K Sports	4.176	6.593	4.900	7.616	−3.112	0.002	0.101	0.008, 0.194	0.528
L Living Things	4.389	6.408	5.711	9.871	−5.967	0.000	0.155	0.062, 0.248	0.544
M Locations	26.359	20.496	24.889	19.908	−2.136	0.033	−0.073	−0.166, 0.02	0.521
N Measurements	44.089	33.885	62.396	41.667	−12.043	0.000	0.477	0.382, 0.571	0.632
O General substances	13.138	12.475	16.960	17.421	−5.955	0.000	0.248	0.155, 0.341	0.57
P Education	4.290	10.262	4.614	8.682	−4.674	0.000	0.034	−0.059, 0.127	0.51
Q Speech Acts	28.168	25.288	24.310	18.601	−2.007	0.045	−0.177	−0.27, −0.084	0.55
S Social Actions	72.781	59.737	73.062	52.393	−2.220	0.026	0.005	−0.088, 0.098	0.501
T Time	34.453	28.926	37.985	28.055	−4.312	0.000	0.124	0.031, 0.217	0.535
W Environment	1.268	3.327	1.879	4.366	−6.058	0.000	0.155	0.062, 0.248	0.544
X Psychology	62.999	43.383	67.294	45.263	−2.335	0.020	0.097	0.004, 0.19	0.527
Y Science/Tech	2.590	6.086	2.690	4.975	−3.317	0.001	0.018	−0.075, 0.111	0.505
Z Names/Grammar	359.010	247.561	389.823	222.817	−4.970	0.000	0.132	0.039, 0.225	0.537
Z99 Out of Dictionary	16.810	17.383	33.909	30.551	−16.818	0.000	0.669	0.574, 0.765	0.682

**Table 3:** Factor Analysis—Total Variance Explained.

Component	Initial Eigenvalues			Extraction Sums of Squared Loadings			Rotation Sums of Squared Loadings		
	Total	% of Variance	Cumulative %	Total	% of Variance	Cumulative %	Total	% of Variance	Cumulative %
1	237,794.144	87.165	87.165	13.060	68.736	68.736	8.049	42.361	42.361
2	24,898.123	9.127	96.291	1.904	10.022	78.758	6.915	36.397	78.758

**Table 4:** Factor Analysis—Rotated component loadings of variables.

Variables	Component	
	1	2
Number of complex (3+ syllable) words	0.922	0.331
Number of unique 3+ syllable words	0.910	0.332
Number of long (6+ characters) words	0.882	0.459
Number of unique long words	0.880	0.433
Wordy items	0.825	0.304
Number of unique words	0.815	0.519
Overused words (x sentence)	0.756	0.273
Number of difficult sentences (more than 22 words)	0.713	0.460
B Medicine/Health	0.686	0.250
Passive voice	0.663	0.211
Z Names/Grammar	0.431	0.901
Z99 Out of Dictionary words	0.465	0.884
X Psychology	0.351	0.820
A general/abstract term	0.524	0.816
Q Speech Acts	0.168	0.775
M Locations	0.192	0.756
S Social Actions	0.374	0.748
T Time	0.363	0.674
N measurements	0.588	0.653

**Table 5:** Area under the receiver operator curve of readability formula and binary classifiers.

Test Result Variable(s)	AUC	Std. Error ^a^	Asymptotic Sig. ^b^	Asymptotic 95% Confidence Interval	
				Lower Bound	Upper Bound
Gunning Fog	0.893	0.008	0.000	0.878	0.908
Flesch Reading Ease	0.882	0.008	0.000	0.866	0.898
SMOG	0.865	0.009	0.000	0.848	0.882
FORCAST	0.865	0.009	0.000	0.848	0.882
Structural Variables Only	0.807	0.009	0.000	0.788	0.825
Semantic Variables Only	0.785	0.010	0.000	0.766	0.804
All variables	0.872	0.008	0.000	0.857	0.888
Logistic Regression	0.863	0.008	0.000	0.847	0.879

^a^. Under the nonparametric assumption; ^b^. Null hypothesis: true area = 0.5.

**Table 6:** Paired-sample *t*-test of differences in area under the ROC curves.

Pairs	Test Result Pair(s)	Asymptotic		AUC Difference	Std. Error Difference ^b^	Asymptotic 95% Confidence Interval	
		z	Sig. (2-Tail) ^a^			Lower Bound	Upper Bound
1	FORCAST vs. Gunning Fog	−4.331	0.00001483 **	−0.0282	0.128	−0.041	−0.015
2	FORCAST vs. SMOG	−0.021	0.98340065	−0.0001	0.132	−0.014	0.014
3	FORCAST vs. Structural Variables	8.320	0	0.0587	0.134	0.045	0.073
4	FORCAST vs. Factor Analysis	−0.865	0.386964	−0.0071	0.129	−0.023	0.009
5	FORCAST vs. All Variables	0.214	0.83031832	0.0019	0.130	−0.016	0.020
6	FORCAST vs. Flesch Reading Ease	−4.630	0.00000365 **	−0.017	0.130	−0.024	−0.010
7	FORCAST vs. Semantic Variables	6.994	0 **	0.0803	0.136	0.058	0.103
8	Gunning Fog vs. SMOG	7.364	0 **	0.028	0.128	0.021	0.036
9	Gunning Fog vs. Structural Variables	11.619	0 **	0.0869	0.130	0.072	0.102
10	Gunning Fog vs. Factor Analysis	2.844	0.000446202	0.0211	0.125	0.007	0.036
11	Gunning Fog vs. All Variables	3.762	0.00016845 **	0.0301	0.126	0.014	0.046
12	Gunning Fog vs. Flesch Reading Ease	2.581	0.00985279	0.0112	0.126	0.003	0.020
13	Gunning Fog vs. Semantic Variables	9.640	0 **	0.1085	0.132	0.086	0.131
14	SMOG vs. Structural Variables	7.736	0 **	0.0589	0.134	0.044	0.074
15	SMOG vs. Factor Analysis	−0.866	0.38625534	−0.0069	0.129	−0.023	0.009
16	SMOG vs. All Variables	0.244	0.8072572	0.0021	0.130	−0.015	0.019
17	SMOG vs. Flesch Reading Ease	−3.750	0.0001769 **	−0.0168	0.130	−0.026	−0.008
18	SMOG vs. Semantic Variables	6.867	0 **	0.0805	0.136	0.057	0.103
19	Structural Variables vs. Factor Analysis	−7.964	0 **	−0.0658	0.132	−0.082	−0.050
20	Structural Variables vs. All Variables	−6.408	0 **	−0.0568	0.132	−0.074	−0.039
21	Structural Variables vs. Flesch Reading Ease	−11.563	0 **	−0.0757	0.132	−0.089	−0.063
22	Structural Variables vs. Semantic Variables	1.831	0.06706809	0.0216	0.138	−0.002	0.045
23	Factor Analysis vs. All Variables	1.202	0.22921297	0.009	0.127	−0.006	0.024
24	Factor Analysis vs. Flesch Reading Ease	−1.330	0.18366955	−0.0099	0.127	−0.024	0.005
25	Factor Analysis vs. Semantic Variables	7.602	0 **	0.0874	0.133	0.065	0.110
26	All Variables vs. Flesch Reading Ease	−2.239	0.02512841	−0.0189	0.128	−0.035	−0.002
27	All Variables vs. Semantic Variables	7.342	0 **	0.0784	0.134	0.057	0.099
28	Flesch Reading Ease vs. Semantic Variables	8.548	0 **	0.0973	0.134	0.075	0.120

^a^. Null hypothesis: true area difference = 0; ^b^. Under the nonparametric assumption, ** P is significant at the adjusted Bonferroni correction 0.00179.

**Table 7:** Sensitivity and specificity of the readability formula under different thresholds.

Formula	Thresholds	Sensitivity	Sensitivity	Formula	Thresholds	Sensitivity	Sensitivity
Gunning Fog	9.7500	0.919	0.685	Flesch Reading Ease	37.5000	0.906	0.690
	9.8500	0.908	0.705		38.5000	0.889	0.713
	9.9500	0.897	0.720		39.5000	0.868	0.744
	10.0500	0.893	0.739		40.5000	0.851	0.764
	10.1500	0.882	0.755		41.5000	0.828	0.788
	10.2500	0.874	0.771		42.5000	0.807	0.811
	10.3500	0.862	0.785		43.5000	0.779	0.825
	10.4500	0.844	0.799		44.5000	0.763	0.845
	10.5500	0.831	0.804		45.5000	0.729	0.856
	10.6500	0.821	0.815		46.5000	0.700	0.871
	10.7500	0.807	0.829		47.5000	0.675	0.879
	10.8500	0.792	0.839		48.5000	0.643	0.891
	10.9500	0.769	0.851		11.9500	0.644	0.866
	11.0500	0.753	0.863		12.0500	0.612	0.881

**Table 8:** Mean and SD of Sensitivity and Specificity of Binary Classifiers.

Variables	Sensitivity Mean (SD)	Specificity Mean (SD)
Semantic Variables Only	0.795 (0.015)	0.762 (0.029)
Structural Features	0.843 (0.011)	0.776 (0.027)
All variables (49)	0.907 (0.012)	0.853 (0.034)
Factor Analysis (19)	0.890 (0.005)	0.860 (0.007)

**Table 9:** Paired-sample *t*-test of differences in sensitivity.

Pairs	Variables	Mean Difference	S.D.	95% Confidence Interval of Difference		*t*	Sig. (2-Tailed)
				Lower	Upper		
Pair 1	Semantic Variables vs. Structural Variables	−0.0478	0.0153	−0.0668	−0.0288	−6.9770	0.0020 **
Pair 2	Semantic Variables vs. Factor Analysis	−0.0744	0.0134	−0.0911	−0.0577	−12.3730	0.0000 **
Pair 3	Semantic Variables vs. All Variables	−0.1116	0.0180	−0.1340	−0.0892	−13.8470	0.0000 **
Pair 4	Structural Variables vs. Factor Analysis	−0.0266	0.0085	−0.0372	−0.0160	−6.9950	0.0020 **
Pair 5	Structural Variables vs.All Variables	−0.0638	0.0182	−0.0864	−0.0412	−7.8450	0.0010 **
Pair 6	Factor Analysis vs. All Variables	−0.0170	0.007	−0.0011	−0.0329	−2.6154	0.0398

** P significant at adjusted Bonferroni 0.00833.

**Table 10:** Paired-sample *t*-test of differences in specificity.

Pairs	Variables	Mean Difference	S.D.	95% Confidence Interval of Difference		*t*	Sig. (2-Tailed)
				Lower	Upper		
Pair 1	Semantic Variables vs. Structural Variables	−0.014	0.047	−0.073	0.045	−0.664	0.543
Pair 2	Semantic Variables vs. Factor Analysis	−0.098	0.028	−0.132	−0.063	−7.890	0.001 **
Pair 3	Semantic Variables vs. All Variables	−0.091	0.045	−0.147	−0.035	−4.520	0.011
Pair 4	Structural Variables vs. Factor Analysis	−0.084	0.027	−0.117	−0.050	−6.927	0.002 **
Pair 5	Structural Variables vs. All Variables	−0.077	0.022	−0.104	−0.050	−7.915	0.001 **
Pair 6	Factor Analysis vs. All Variables	0.007	0.034	−0.036	0.049	0.434	0.687

** P significant at adjusted Bonferroni 0.00833.

## Data Availability

The raw data supporting the conclusions of this article will be made available by the authors, without undue reservation.
